# The impact of a targeted education package on the knowledge, attitudes, and utilisation of patient reported outcome measures amongst chiropractors in Australia

**DOI:** 10.1186/s12998-022-00450-4

**Published:** 2022-10-14

**Authors:** Natalie Clohesy, Anthony Schneiders, Gaery Barbery, Steven Obst

**Affiliations:** 1 College of Health Sciences, School of Health, Medical and Applied Sciences, Central Queensland University, Bundaberg, QLD Australia; 2 School of Psychology, Griffith University, South Brisbane, QLD Australia

**Keywords:** Patient reported outcome measures, Chiropractic, Knowledge to action, New World Kirkpatrick Model, Behaviour change

## Abstract

**Background:**

Patient Reported Outcome Measures (PROMs) have been shown to be valid and reliable indicators of health status and treatment outcomes, however, the current knowledge, understanding, and utilisation of PROMs within the Australian Chiropractic profession is limited. This study used the New World Kirkpatrick Model (NWKM) to evaluate whether an online PROM education package could improve knowledge, confidence, attitude, and utilisation of PROMs by chiropractors in Australia.

**Methods:**

A longitudinal cohort interventional study of chiropractors in Australia. The recruitment phase and data collection period occurred from November 2020 – May 2021. Participants completed three online surveys two weeks before, four weeks after, and 12 weeks after receiving an online education package that included ten evidence-based region-specific modules on PROMs. Survey questions were grouped into five subthemes for analysis according to the NWKM levels: (1) Reaction; (2) Learning - knowledge; (3) Learning – confidence; (4) Behaviour – attitude; (5) and Behaviour - utilisation).

**Results:**

Of the 116 participants that enrolled in the study, 43 completed all three survey and were included in the analysis. There was very positive reaction to the education package with mean response scores (1–5 Likert scale) for the reaction questions ranging from 3.75 to 4.43. There was a small, but significant, increase in knowledge (out of 32) at four weeks (24.3 ± 6.1) and 12 weeks after receiving the education package (27.2 ± 5.5), compared to baseline (27.4 ± 5.1). There was no effect of the intervention on clinician confidence or attitude towards PROMs. Utilisation of function- and pain-related PROMs did not change after the intervention. There was a small and significant (p < 0.05) increase in utilisation of health-related PROMs 12 weeks after the intervention.

**Conclusion:**

Despite modest improvements in knowledge, which were retained 12 weeks after the educational package was provided, there was no evidence that participant confidence, attitude, or utilisation of PROMs changed because of the intervention. While the respondents’ have positive attitudes and beliefs regarding PROMs use, further education surrounding the clinical translation process into clinical practice is required. Caution is advised when interpreting these findings due to the low participant response and completion rate with the potential for selection bias and the inability to generalise the results.

**Supplementary information:**

The online version contains supplementary material available at 10.1186/s12998-022-00450-4.

## Background

Patient reported outcome measures (PROMs) are validated questionnaires and survey instruments used to measure the status of a patient’s health condition using information that comes directly from the patient [1, 2]. PROMs were initially used as epidemiological surveys to identify patterns of symptomatology or health status, however, they have evolved to be tools that can also be used to support clinical practice and research [3]. In the clinical setting, PROMs can assist the decision-making process, improve therapist-patient communication, monitor treatment progress, and/or facilitate patient-centered care [4–7]. PROMs are therefore considered an integral part of evidence-based health care and therefore it is important that clinicians are equipped with the requisite skills and knowledge needed to apply PROMs in clinical practice.

A recent survey of chiropractors in Australia found that 72% of respondents reported using PROMs for the management of low back pain (LBP) [8]. Similar rates of PROM utilisation were reported for physiotherapists in the United Kingdom, where 72% of physiotherapists, and 71% of physiotherapy practices, routinely used PROMs [9]. Despite these relatively high rates, it has been suggested there is a lack of knowledge and understanding of PROMs amongst chiropractors, which may limit the full clinical implementation of PROMs and their potential benefits [5, 8–11]. For example, a survey of chiropractors in Australia found that 19% of respondents reported that a lack of knowledge regarding PROMs prevented clinical implementation, while time required to administer and score PROMs in a clinical setting (17%) was also a barrier to their utilisation [8]. The results of studies by Holmes, Bishop [5] and Antunes, Harding [12] mirrored these findings, suggesting that a clinician’s lack of PROM knowledge was a significant barrier to implementation.

Although chiropractors are increasingly encouraged to use PROMs [13], a lack of knowledge transfer from literature, education, and training into clinical practice are likely barriers to their successful implementation into clinical practice [5, 14]. Furthermore, even though chiropractors place high value on the importance of PROMs in clinical practice, these beliefs do not appear to translate to increased frequency of PROM usage, suggesting other factors, including knowledge, may be central to changing behaviors [15].

To improve the transfer of knowledge to practice, a number of theories, models and frameworks have been developed [16]. Within allied health settings, the most highly cited and accepted model is the Knowledge to Action framework (KTA) [17–20] created by Graham and Tetroe [21]. KTA is a conceptual framework made up of two distinct components - knowledge creation and the action cycle. The knowledge creation phase is represented as a tunnel and the information becomes more refined and specific as it passes through this phase to uncover the most valid and useful knowledge [22]. The action phase is a cycle leading to implementation or application of knowledge uncovered in the knowledge creation phase. During this step, adapting knowledge to meet the needs within particular contexts or populations occurs [23].

The aim of this study was to evaluate the impact of an educational package developed using the New World Kirkpatrick Model (NWKM) according to four levels; reaction, learning, behaviour and results [24]. The implementation was assessed using KTA framework to improve PROM knowledge and utilisation in a cohort of chiropractors in Australia. We hypothesised that a tailored educational package intervention would increase participant knowledge of PROMs, which would translate to a positive change in attitude and frequency of PROM utilisation in clinical practice.

## Methods

### Study design

A longitudinal interventional cohort survey with repeated measures was used to gain an understanding of the respondent’s knowledge, attitudes, and utilisation of PROMs in the Chiropractic profession of Australia. An online educational package was designed, delivered and the findings evaluated using the New World Kirkpatrick Model (NWKM) [24–26] using three of the four key themes of the model: reaction, learning, and behaviour. The study was conducted over 7 months and included three online surveys administered at specific timepoints using the Qualtrics software. Full ethical approval was granted by the Human Research Ethics Committee of Central Queensland University, reference number 0000022391.

### Participants

Chiropractors who were current members of the Australian Chiropractic Association (ACA) or Chiropractic Australia (CA) were invited to participate in the study via member emails and advertisement in the association’s newsletters. The sample population consisted of 3,215 ACA members and 1,163 CA members, totaling 4,378 potential participants, representing the majority of Chiropractors registered and practicing in Australia in 2020 [27]. The recruitment phase and data collection period occurred from November 2020 – May 2021.

### Longitudinal surveys

Participants were surveyed on three occasions: (1) two weeks before the educational package (Survey 1); (2) four weeks after receiving the educational package (Survey 2); and (3) 12 weeks after receiving the educational package (Survey 3). Survey 1 collected demographic information including age, sex, country of graduation, highest level of education, in addition to the participant’s knowledge and attitudes about PROMs and their preferred mode of delivery (e.g., audio, audio-visual or transcript) for the educational package. Survey 2 and 3 repeated the knowledge and attitude questions from Survey 1, while omitting the demographic questions, and including items assessing the participant’s reaction to the educational package.

### Educational package

The content and design of the educational package was informed by data obtained from Survey 1, and therefore, being co-designed [28]. Respondents were given multiple-choice questions with options of content to be included in the educational package. There was also an open text box to allow them to formulate their own answers and request specific information to be included, which is recommended when using survey methods [29]. Survey 1 also asked the respondents about their preference of mode for the delivery of the educational package. Of the 116 respondents, 81% were very likely/likely to view via text (electronic), 57% via audio-video format, 55% via text (paper), and 35% via audio. Given the spread of preferred formats the educational package was adapted and made available via for all three modes – audio-visual, audio and text [30].

When asked about what topics should be included in the package, most respondents requested ‘Examples of the best PROMs for major conditions’ (85%), ‘How PROMs can be used in Chiropractic clinical practice (83%)’, and ‘Explanation of categories of PROMs (80%)’. The subsequent educational package consisted of 10 modules: 1) introduction; 2) background of PROMs; 3) PROMs in Chiropractic practice; 4) non-location specific PROMs; 5) PROMs for headache, whiplash, temporomandibular joint and dizziness; 6) PROMs for cervical spine; 7) PROMs for thoracic spine; 8) PROMs for lumbar spine; 9) PROMs for upper extremity; and 10) PROMs for lower extremity. For modules 4–10, the following topics were included (where available and/or appropriate) for each example PROM: category, overview, scoring, area of assessment, method of delivery, and clinical condition(s). The duration of the ten audio-visual and audio modules ranged from approximately one to fifteen minutes. The full educational package is available in all formats.

### Data analysis

Survey data was exported from Qualtrics software and analysed using the Statistical Package for the Social Sciences (SPSS version 28). A one-way repeated measure analysis of variance was used to assess the effect of time (two weeks before, four weeks after, and 12 weeks after) on knowledge scores, with post-hoc paired t-tests used to compare time points. For non-parametric data obtained from Likert responses, the effect of time was assessed using the Friedman test with post-hoc Wilcoxon signed-rank test used to compare time points. All post-hoc tests were corrected for multiple comparisons using Bonferroni alpha corrections. To aid interpretation and discussion survey questions were thematically grouped a-priori according to each level within the NWKM - reaction, learning, and behavior (see Supplementary Table 1). The correlation between response data of thematically grouped questions were assessed using Cronbach’s alpha with α scores > 0.7 considered satisfactory [31].

## Results

A total of 116 participants completed Survey 1 and were provided with the educational package and an invitation to complete Survey 2 and 3. Of these participants, 52 completed Survey 2 (47% retention) and 43 completed Survey 3 (37% retention from those who completed the initial survey). A summary of participant characteristics is presented in Table 1.

**Table 1:** Descriptive summary of participant characteristics for each survey

Survey number	n	Male	Female	Most common age bracket	Most common years practice	Most common country of graduation	Principle	Associate	Holds a post-graduate qualification
S1	116	61%	39%	35–39 years (23%)	10–14 years (31%)	Australia (89%)	79%	21%	25%
S2	52	59%	41%	35–39 years (22%)	10–14 years (28%)	Australia (90%)	91%	9%	21%
S3	43	63%	37%	35–39 years (23%)	10–14 years (26%)	Australia (88%)	93%	7%	25%

### Level 1—reaction to the educational package

Participant reaction to the educational package was evaluated using eight questions from Survey 3 (n = 43) grouped according to four subthemes – knowledge, behaviour, relevance, and satisfaction (Table 2). Most respondents viewed the educational package favorably with mean response scores ranging from 3.75 (‘I would recommend changes to my practice procedures after viewing this educational package’) and 4.43 (‘The educational package was effective at increasing my knowledge’) on a 5-point Likert scale. Most participants were either somewhat or strongly satisfied with the overall educational package (81%), and either strongly or somewhat agreed that the educational package improved their knowledge of PROMs (87%). Similarly, 72% of participants were satisfied with the duration of the package and 99% felt that the package was relevant to their needs, with 88% reporting they would recommend it to their colleagues. The correlation between subtheme questions ranged between α = 0.71 and 0.91.

**Table 2:** Reaction to the education package

		Frequency of respondents (%)				
Survey question	Mean score (1–5)	Strongly disagree (1)	Somewhat disagree (2)	Neutral (3)	Somewhat agree (4)	Strongly agree (5)
Knowledge						
The education package was effective at increasing my knowledge	4.43	0%	0%	12%	35%	53%
Did the education tool improve your knowledge of PROMs?	4.32	0%	0%	14%	42%	44%
Behaviour						
I would recommend changes to my practice procedures after viewing this education package.	3.75	5%	5%	21%	49%	21%
I would recommend this education package to a colleague.	4.39	0%	0%	12%	39%	49%
Relevance						
The education package was relevant to my needs.	4.23	0%	2%	14%	42%	42%
The education package matched my learning style.	4.23	0%	2%	14%	42%	42%
Satisfaction						
I was satisfied with the overall quality of the education package.	4.34	0%	2%	16%	28%	53%
I was satisfied with the duration of the education package	4.07	0%	0%	5%	33%	39%

### Level 2—learning (knowledge about PROMs)

Participant knowledge of PROMs was evaluated in each survey using 32 questions that covered topics including PROM definitions, categories, and specific examples. The mean (SD) knowledge scores (out of 32) for Survey 1, 2, and 3 were 24.2 (6.1), 27.2 (5.5), and 27.4 (5.1), respectively. The one-way repeated measured analysis of variance (n = 43) revealed a significant effect of time on knowledge (*F*_41,1_ = 21.198, p < 0.001). Post-hoc t-tests indicate a significant improvement in knowledge from Survey 1 to Survey 2 (mean difference ± 95%CI = 3.0 ± 2.5, p = 0.013) and Survey 1 to Survey 3 (mean difference ± 95%CI = 3.1 ± 1.7, p < 0.001), with no significant difference in knowledge between Survey 2 and 3 (p = 1.00).

### Level 2 learning—confidence in understanding and using PROMs

The related samples Friedman’s analysis of variance by ranks test (n = 39) revealed no significant effect of time on clinician confidence in: (1) understanding what a PROM is (χ^2^ (2) = 0.607, p = 0.738); (2) understanding the significance of using PROMs (χ^2^ (2) = 0.157, p = 0.924); (3) recognising when to apply PROMs (χ^2^ (2) = 0.157, p = 0.924); (4) implementing PROMs (χ^2^ (2) = 0.171, p = 0.918); or (5) knowing what patient reported measures are available (χ^2^ (2) = 0.639, p = 0.726). The correlation between the questions α = 0.899. The mean ranks for each question at each time point are included in Table 3.

**Table 3:** Change in participant confidence using patient reported outcome measures before and after receiving the education package

Survey question	Survey 1 Mean rank	Survey 2 Mean rank	Survey 3 Mean rank
How confident are you understanding what a patient reported outcome measure is?	1.95	2.06	1.99
How confident are you understanding the significance of patient reported outcome measures?	2.08	1.96	1.96
How confident are you recognising when to use patient reported outcome measures?	2.04	1.99	1.97
How confident are you implementing patient reported outcome measures?	2.01	2.03	1.96
How confident are you knowing what patient reported outcome measures are available?	1.92	2.01	2.06

### Level 3—behaviour (attitude towards PROMs)

There was no significant effect of time on how influential PROMs were to the treatment plan and patient management (χ^2^ (2) = 3.644, p = 0.162) (see Table 4). There was a significant effect of time on whether health professional should use PROMs using valid and reliable tools (χ^2^ (2) = 6.982, p = 0.03), however, the post-hoc Wilcoxon rank-tests revealed no significance difference between Survey 1 and 2 (Z = 1.670, p = 0.095) or between Survey 1 and 3 (Z = 1.485, p = 0.138). There was no effect of time on clinician attitudes to PROMs based on the remaining statements: (1) ‘PROMs enable you to get a better understanding of your patient’s progress’ (χ^2^ (2) = 1.627, p = 0.443); (2) ‘The use of validated PROMs is clinically helpful in an increasing medicolegal environment’ (χ^2^ (2) = 1.853, p = 0.396); (3) ‘The use of patient reported outcome measures could be helpful in justifying ongoing treatment to third parties’ (χ^2^ (2) = 4.680, p = 0.096); (4) ‘My patients are all different; therefore patient reported outcome measures would not be useful’ (χ^2^ (2) = 0.628, p = 0.731); (5) ‘Available patient reported outcome measures are unsuitable for the type of patients I treat’ (χ^2^ (2) = 2.086, p = 0.352); (6) ‘If I had more time, I would be interested in using patient reported outcome measures’ (χ^2^ (2) = 0.609, p = 0.738); (7) ‘I do not see the use of patient reported outcome measures as a priority’ (χ^2^ (2) = 2.385, p = 0.304); (8) ‘Patient reported outcome measures are unpopular with patients’ (χ^2^ (2) = 1.681, p = 0.432); (9) ‘Patient satisfaction is the most important outcome’ (χ^2^ (2) = 4.978, p = 0.083); (10) ‘I do not know enough about patient reported outcome measures to feel comfortable/confident using them’ (χ^2^ (2) = 0.828, p = 0.661); 11) ‘The patient discontinuing treatment puts me off using patient reported outcome measures’ (χ^2^ (2) = 0.023, p = 0.998); 12) ‘There is no need to change from the way that we have to assess/assessed patients’ (χ^2^ (2) = 2.141, p = 0.343); 13) ‘If I had to use patient reported outcome measures, I would prefer to choose which ones I used’ (χ^2^ (2) = 2.049, p = 0.359); 14) ‘Access to information about patient reported outcome measures is limited in my work environment’ (χ^2^ (2) = 1.162, p = 0.559); and 15) ‘It is not necessary to measure functional outcomes’ (χ^2^ (2) = 0.667, p = 0.717). The correlation between the questions α = 0.655.

**Table 4:** Change in participant attitude towards patient reported outcome measures before and after receiving the education package

Survey question	Survey 1 Mean rank	Survey 2 Mean rank	Survey 3 Mean rank
How influential are patient reported outcome measures to your treatment plan and patient management?	2.01	1.85	2.14
Health professionals should use patient reported outcome measures to monitor treatment outcomes using valid and reliable tools.	1.81	2.17*	2.03
Patient reported outcome measures enable you to get a better understanding of your patient’s progress	1.94	2.12	1.95
The use of validated patient reported outcome measures is clinically helpful in an increasing medicolegal environment.	2.12	1.92	1.96
The use of patient reported outcome measures could be helpful in justifying ongoing treatment to third parties	2.15	1.96	1.88
My patients are all different; therefore, patient reported outcome measures would not be useful	2.04	2.04	1.92
Available patient reported outcome measures are unsuitable for the type of patients I treat	2.10	2.04	1.86
If I had more time, I would be interested in using patient reported outcome measures	1.92	2.05	2.03
I do not see the use of patient reported outcome measures as a priority	2.14	1.91	1.95
Patient reported outcome measures are unpopular with patients	2.13	1.96	1.91
Patient satisfaction is the most important outcome	2.06	1.78	2.15
I do not know enough about patient reported outcome measures to feel comfortable/confident using them	2.00	1.92	2.08
The patient discontinuing treatment puts me off using patient reported outcome measures	2.00	1.99	2.01
There is no need to change from the way that we have to assess/assessed patients	1.86	2.08	2.06
If I had to use patient reported outcome measures, I would prefer to choose which ones I used	2.13	1.97	1.90
Access to information about patient reported outcome measures is limited in my work environment	2.05	2.06	1.88

### Level 3—behaviour (Utilisation of PROMs)

There was no significant effect of time on the frequency of pain-related (χ^2^ (2) = 0.192, p = 0.909) or functional-related (χ^2^ (2) = 3.69, p = 0.158) PROM use (Table 5). There was, however, a significant effect of time on the frequency of health-related PROM use (χ^2^ (2) = 8.310, p = 0.016). Post-hoc Wilcoxon signed-rank tests revealed a significant increase in the mean rank score for health-related PROM use from Survey 1 (1.79) to Survey 3 (2.01) (Z = 2.707, p = 0.007, ES = 0.41) with 14 positive differences, 3 negative differences, and 27 ties (Table 3). There was no difference in the mean rank score between Survey 1 to Survey 2 (Z = 0.125, p = 0.901), with 10 positive differences, 7 negative differences, and 35 ties. When asked about whether the educational package increased their use of patient reported outcome measures (PROMs) in clinical practice, 39% of participants in Survey 2 answered ‘yes’ (41% No, 17% Unsure), compared to 56% in Survey 3 (28% No, 16% Unsure).

**Table 5:** Changes in participant utilisation of patient reported outcome measures before and after receiving the education package

Survey question	Survey 1 Mean rank	Survey 2 Mean rank	Survey 3 Mean rank
How often on average do you use PROMs in your everyday practice (ANY)	2.03	2.00	1.97
How often on average do you use PROMs in your everyday practice (PAIN)	2.01	1.96	2.03
How often on average do you use PROMs in your everyday practice (FUNCTIONAL)	1.86	1.95	2.19
How often on average do you use PROMs in your everyday practice (HEALTH)	1.79	2.01	2.19*

*Statistically different to Survey 1 (p < 0.05)

## Discussion

This longitudinal study evaluated the impact of an educational package developed and implemented using the KTA framework, and which aimed to increase PROM knowledge and utilisation in a small cohort of chiropractors in Australia. The results are presented and discussed according to three of the four levels of the NWKM – reaction, learning, and behavior [32]. Overall, the reaction to the educational package was very positive, with most participants noting improved knowledge and a high level of satisfaction with the material. Although there was a significant improvement in knowledge after receiving the educational package, which was retained at 12 weeks, these changes were small and did not translate to improvements in confidence, attitude, or frequency of PROM usage. The findings suggest that changes in knowledge alone may be insufficient to influence the frequency of PROM utilisation by chiropractors.

### Level 1—reaction

It is important to evaluate the participant’s reactions to the course or training when evaluating the success of the educational intervention [33]. Overall, the findings confirmed a positive reaction to the educational package. Most respondents reported that the intervention was favourable, engaging, and relevant to their needs. These findings could therefore be important to create clinical behaviour change as Oreg, Vakola [34] found that organizational and behaviour change are not well implemented unless there is a positive reaction.

Survey 1 included items which allowed respondents to communicate directly with the content creators and inform design and content of the educational package [28, 35]. Importantly, 98% of those surveyed felt that the educational package they engaged with was highly relevant to their needs and matched their learning style; the latter of which has been associated with improved learning outcomes in educational [36]. Furthermore, 81% of the survey respondents were either somewhat or strongly satisfied with the overall educational package and 88% stated that the educational package was effective at increasing their knowledge and they would recommend it to their colleagues. Interestingly, only 70% of the respondents noted that they would recommend changes to their practice procedures after viewing the educational package. While research suggests that participant reaction to learning is important such measures may not correlate to how much participants learnt, or whether their behaviour changed [37–40]. For example, a recent study of acupuncturists noted that while the reaction to the training is important, levels 2 (learning) and 3 (behaviour) of the NWKM are the most relevant to determine the success of the program [41].

### Level 2—learning

Level two of the NWKM is concerned with determining the degree of learning, knowledge, confidence, and skill acquisition because of training [26, 32, 42, 43]. To assess knowledge, evaluation of the participant’s recall, understanding and application of the learning should be measured [44]. Recall and understanding were assessed using three questions included in each survey and covered a range of topics including the definition of PROMs, categories of PROMs, and the identification of PROMs examples using multiple-choice questions. The results show a significant improvement in knowledge scores between survey 1 and survey 2, which were retained at survey 3, suggesting the intended learning outcomes were achieved and the educational package was successful [45, 46]. Nevertheless, the improvements in knowledge were modest (~ 10%), and while statistically significant, may be insufficient to affect a change in participant behavior and attitude towards PROMs. Despite a recent survey of chiropractors in Australia suggesting that improving clinician understanding of PROMs and why/when to use PROMs would improve utilisation rates, our study, together with others, suggest that knowledge, in and of itself, may not be sufficient to change behavior [47, 48]. Similarly, confidence of the learner, which has been suggested to be a gap between learning and behaviour [49], was also not changed as a result of the educational package. We found no difference in confidence scores between survey 1, and surveys 2 and 3. Research suggests that motivation and confidence are key determinants to create behavioral change [50]. Boyce, Robertson [51] further suggest that building confidence is important if intentions are to be translated into behaviour change. Nevertheless, while confidence may bridge the gap between knowledge and behavior, improving knowledge alone seems insufficient to change confidence. Overall, this study suggests that the provision of an online educational package aimed to improve knowledge of PROMs in chiropractors had no effect on their confidence in understanding what PROMs were available or when and/or how to use them.

### Level 3—behaviour

Level 3 of the NWKM evaluates to what extent the newly acquired knowledge or skills have been practically applied [42, 52]. This level also assesses the degree to which the participants apply what they learned and modified their behaviour based on the intervention [53]. In the current study we evaluated behavior change using questions related to two subthemes: (1) attitude toward PROMs; and (2) frequency of PROM utilisation. Participant’s attitude toward PROMs were evaluated using 16 questions, which covered a variety of attitudinal aspects relating to the importance and influence of PROMs in clinical practice. Of the 16 questions, only one question was significantly different after the provision of the educational package. Participants more strongly agreed with the statement: ‘Health professionals should use patient reported outcome measures to monitor treatment outcomes using valid and reliable tools’ in survey 2, compared to survey 1. The educational package may have highlighted the arrival of the “era of accountability” with more pressure placed on health care providers to provide treatment evidence [54]. The last decade has seen an increasing focus and interest not only in a patients symptoms, but also in documenting the patient experience and their interactions with healthcare providers [55]. Nevertheless, the overall results do not support a change in clinician attitudes toward PROMs after receiving the educational package. Whilst difficult to determine, it is likely that respondents already had a very favorable view towards PROMs and so there may have been a ceiling effect limiting the impact of the educational package on participant attitudes. Previous studies do show that most chiropractors have a favorable view of PROMs [5, 15], however, these views do not necessarily correlate to higher levels of usage [15].

Although 70% of the respondents reported that they would recommend changes to their practice procedures after viewing the educational package, the data did not support that those changes occurred. Furthermore, despite most respondents acknowledging the importance of PROMs and the need for clinical change to include PROMs, the clinical implementation did not increase after receiving the educational package. Except for health-related PROMs, the reported frequency of PROM utilisation did not change from survey 1 to surveys 2 and 3. We found a small but significant increase in the reported use of health-related PROMs from survey 1 to survey 3. Although there was a similar trend toward increased use of functional PROMs, these differences were not significant. These improvements may be explained, at least in part, by two factors. First, the current study, as well as previous studies [8], show that functional and health-related PROMs are less frequently used by chiropractors in clinical practice, compared to pain-related PROMs, and so their frequency of use may be more likely changed with an educational package that promotes awareness of these PROMs. Secondly, and further to this point, the educational package was specifically designed to address these practice gaps by providing a comprehensive summary of numerous health- and functional-related PROMs relevant to each body region. It is possible that much of this content may have been perceived as ‘new and novel’ and therefore may be more likely to elicit a measurable change in behaviour, compared to the more commonly used pain-related PROMs.

Overall, the educational package did not affect a measurable change in participant’s confidence, attitude, or frequency of PROM utilisation. Although we used the KTA framework to design the content of the educational package, we did not include any specific strategies to promote translation, but rather relied on knowledge to drive behavior change. Clearly, knowledge alone is not sufficient to affect behavioral change. Although knowledge has been consistently identified as a barrier/facilitator to PROM utilisation in healthcare, multiple other barriers exist which were not addressed in the current study and may be more influential in changing behavior. Time was the most reported barrier to PROM usage by chiropractors [5, 8]. Although the educational package provided links to source material there were no specific implementation strategies included in the package that may have eased the time burden of using PROMs in clinical practice. The provision of all relevant PROMs to chiropractic care, including background information, via a single electronic application may be a future strategy to better align PROM knowledge with PROM access, and reduce the time the burden of accessing PROMs in a clinical setting.

It is possible that if this study had continued over a longer period of time, this may have increased the likelihood of measuring behavioral change. Axtell, Maitlis [56] found that the amount of learning transferred into practice one month post intervention was a strong predictor for learning transferred one year post intervention. According to Kirkpatrick D [57] the most accurate time to evaluate behaviour change is at least three months after the training was applied, although literature suggests that behaviour change may not occur until six months, to enable learners sufficient time to put their new skills into practice [58]. Therefore, a longer-term follow-up (up to 12 months) may have been needed to identify whether the short-term changes in knowledge changed behaviour.

#### Future directions

The findings from this study have implications for future research in this field, specific to clinical implementation and for future contributions to literature.

A modification to the current education package could include the addition of a module specific to implementation of PROMs in clinical practice, e.g. the addition of a multi-modal (video, text and audio files) module outlining the exact steps to apply PROMs would be warranted. Using more technology-based apps, electronic links to PROMs (copyright pending) would possibly assist chiropractors by making PROMs more accessible and less time consuming. The education package could be offered to Chiropractors through chiropractic professional organisations/governing bodies as continuing professional development and ensure chiropractors are receiving the most up to date information. Additionally, the education package could be embedded into Chiropractic universities curriculum to ensure graduating Chiropractors are proficient in the implementation of PROMs at the beginning of their clinical careers.

## Limitations

In addition to the short follow-up period, there are several other limitations to this study. First, the low response rate to the cohort survey may limit the strength and generalisability of the findings. Although online surveys are a common research method, their success, particularly amongst health care professionals has been questioned [59]. Cunningham, Quan [59] imply that low survey response rates are common within the health care profession. Another limitation is bias, which is an inherent issue in the design of surveys [60]. Although the authors aimed to reduce selection bias and population bias by inviting all members of Australia’s main chiropractic associations to participate, an unintentional responder bias may have occurred. Due to the low response rate, the survey respondents may not be representative of the entire chiropractic profession in Australia. A further limitation of this study was that there was no control group. Additionally, given the substantial drop-out between survey 1 and survey 2, it is possible the participants who completed all three surveys may have already had favorable views of PROMs, and relatively high utilisation rates, and so there may have been a potential ceiling effect associated with the educational package that affected changes in confidence, attitude, and frequency of PROM usage.

## Conclusion

An online educational package delivered to chiropractors in Australia was effective at improving knowledge of PROMs, including what PROMs are and why, how, and when they should be used in clinical practice. Despite modest improvements in knowledge, which were retained 3-months after the educational package was provided, there was no evidence that participants confidence, attitudes, or frequency of PROM use changed because of the intervention. The study findings suggest that knowledge of PROMs alone may be insufficient to change the frequency of their use in clinical practice by chiropractors in Australia. However, caution is advised when interpreting these findings due to the low participant response and completion rate with the potential for selection bias and the inability to generalise the results.

## Electronic supplementary material

Below is the link to the electronic supplementary material.

Supplementary Material 1

## Data Availability

Not applicable.
