# Supplementary material for: The impact of a targeted education package on the knowledge, attitudes, and utilisation of patient reported outcome measures amongst chiropractors in Australia
Source: Chiropr Man Therap. 2022 Oct 14;30:44. doi: 10.1186/s12998-022-00450-4 (PMC9569072; doi:10.1186/s12998-022-00450-4)
Supplement: Supplementary file 1 — Supplementary Material 1 [file 12998_2022_450_MOESM1_ESM.docx]

Table 1. Survey questions corresponding to the three levels of the New World Kirkpatrick Model

|  | Survey 1 | Survey 2 | Survey 3 |
| --- | --- | --- | --- |
| **Level 1 - Reaction** | | | |
| The education package was effective at increasing my knowledge package |  |  |  |
| Did the education package improve your knowledge of PROMs? |  |  |  |
| I would recommend this education package to a colleague |  |  |  |
| I would recommend changes to my practice procedures after viewing this education package. |  |  |  |
| The education package matched my learning style |  |  |  |
| The education was relevant to my needs |  |  |  |
| I was satisfied with the overall quality of the education package |  |  |  |
| I was satisfied with the duration of the education |  |  |  |
| Do you have any comments or feedback about patient reported outcome measures (PROMs) or the patient reported outcome measures (PROM) education package? |  |  |  |
| **Level 2 – Learning** | | | |
| Which definitions from the list below best describes a patient reported outcome measure (PROM) |  |  |  |
| From the list below please answer whether the option represents a CATEGORY of a patient reported outcome measure (PROM) |  |  |  |
| From the list below please answer whether the options are EXAMPLES of a patient reported outcome measure (PROM) |  |  |  |
| I do not know enough about patient reported outcome measures to feel comfortable/confident using them |  |  |  |
| Understanding what a patient reported outcome measure (PROM) is. |  |  |  |
| Understanding the significance of patient reported outcome measure (PROM) use. |  |  |  |
| Recognising when to apply patient reported outcome measures (PROMs) in practice. |  |  |  |
| Implementing patient reported outcome measures (PROMs). |  |  |  |
| Knowledge of what patient reported outcome measures (PROMs) are available. |  |  |  |
| I would be interested in learning more about patient reported outcome measures |  |  |  |
| **Level 3 - Behaviour** | | | |
| When do you use PROMs? |  |  |  |
| How influential are patient reported outcome measures to your treatment plan and patient management? |  |  |  |
| How often do you use PROMs? |  |  |  |
| I would recommend changes to my practice procedure after viewing this education package |  |  |  |
| I am interested in using clinical outcome measures in my practice |  |  |  |
| Health professionals should use patient reported outcome measures to monitor treatment outcomes using reliable and valid tools |  |  |  |
| Patient reported outcome measures enable you to get a better understanding of your patients’ progress |  |  |  |
| The use of validated patient reported outcome measures is clinically helpful in an increasing medicolegal environment |  |  |  |
| The use of patient reported outcome measures could be helpful in justifying ongoing treatment to third parties |  |  |  |
| My patients are all different; therefore, patient reported outcome measures would not be useful |  |  |  |
| Available patient reported outcome measures are unsuitable for the type of patients I treat |  |  |  |
| I do not see the use of patient reported outcome measures as a priority |  |  |  |
| Patient reported outcome measures are unpopular with patients |  |  |  |
| Patient satisfaction is the most important outcome |  |  |  |
| The patient discontinuing treatment puts me off using patient reported outcome measures |  |  |  |
| There is no need to change from the way that we have assess/assessed patients |  |  |  |
| If I had to use patient reported outcome measures, I would prefer to choose which ones I used |  |  |  |
| Access to information about patient reported outcome measures is limited in my work environment |  |  |  |
| It is not necessary to measure functional outcomes |  |  |  |
| Did the education tool increase your use of patient reported outcome measures (PROMs) in practice? |  |  |  |
| Was the education package useful to assist you to better understand and apply patient reported outcome measures (PROMs) in practice? |  |  |  |
